# Tool manipulation by rats (*Rattus norvegicus*) according to the position of food

**DOI:** 10.1038/s41598-017-06308-7

**Published:** 2017-07-20

**Authors:** Akane Nagano, Kenjiro Aoyama

**Affiliations:** 1 Graduate School of Psychology, Doshisha University, 1-3, Tatara Miyakodani, Kyotanabe, 610-0394 Japan; 2 Faculty of Psychology, Doshisha University, 1-3, Tatara Miyakodani, Kyotanabe, 610-0394 Japan

## Abstract

Tool-use behaviour has been observed in nonhuman animals in the wild and in experimental settings. In the present study, we investigated whether rats (*Rattus norvegicus*) could manipulate a tool according to the position of food to obtain the food in an experimental setting. Eight rats were trained to use a rake-shaped tool to obtain food beyond their reach using a step-by-step protocol in the initial training period. Following training, the rake was placed at the centre of the experimental apparatus, and food was placed on either the left or right side of the rake. Rats learned to manipulate the rake to obtain food in situations in which they could not obtain the food just by pulling the rake perpendicularly to themselves. Our findings thus indicate that the rat is a potential animal model to investigate the behavioural and neural mechanisms of tool-use behaviour.

## Introduction

St Amant and Horton^1^ provided the following definition of tool-use. Tool-use is the exertion of control over a freely manipulable external object to generate a mechanical dynamic interaction between the user and an object or to affect the transfer of information between the user and the environment. Tool use is indispensable for humans, and it is an important component of the survival and fitness of several non-human species as well^2^. For instance, wild woodpecker finches (*Cactospiza pallida*) can hold a twig or cactus spine in their beak, insert it into an opening in the bark of a tree and pry out arthropods as a source of food^3^. In the wild, chimpanzees (*Pan troglodytes*) can hold a stick with their hand and dip it into and around the entrances of underground ant nests to obtain ants for consumption^4^.

In experimental settings, some studies have reported that human infants and nonhuman animals can be trained to use a rake-shaped tool to obtain a toy or food beyond their reach in situations in which they cannot obtain the reward by pulling the tool itself towards them. In previous studies, 14–22-month-old human infants were observed to use a rake-shaped tool to obtain a toy beyond their reach^5–8^. A shared feature of these studies is that the subject was required to manipulate a rake-shaped tool to obtain a reward beyond its reach via a step-by-step protocol. In these studies, various conditions for the arrangement of the rake and the toy were adopted, and the distance between the rake and toy was gradually extended. These studies reported that infants aged 18 months and older could move the rake laterally before pulling on it to obtain a toy under conditions in which the toy was placed to the side of the rake^5–9^. Similar tool-use tasks have also been carried out by Japanese macaques (*Macaca fuscata*)^10–19^, rhesus monkeys (*Macaca mulatta*)^20^, common marmosets (*Callithrix jacchus*)^21, 22^, and degus (*Octodon degus*)^23, 24^. These studies of nonhuman animals show that subjects could be trained to manipulate a rake-shaped tool laterally before pulling on it, allowing them to obtain a food item beyond their reach in situations in which they could not obtain the food by pulling the tool perpendicularly to themselves without further manipulation. However, no research has thus far been conducted on whether rats (*Rattus norvegicus*) can manipulate tools to obtain food in such a manner.

Some studies have reported that subjects could not actually obtain the reward using the rake at the beginning of the training, when the reward was placed at the side of the rake^5–8, 14, 21–24^. For instance, Fagard *et al*.^5^ observed that human infants touched or pushed the toy with the rake but failed to obtain the toy. Okanoya *et al*.^24^ reported that degus waved the rake around the food for a short period of time but failed to obtain the food at the beginning of training. Other studies did not report performance at the beginning of the training^9–13, 15–20^. However, it cannot be concluded that subjects did not understand that the rake required manipulation in the direction of the reward and not in the direction opposite that of the reward to obtain it. It is possible that subjects may have understood the appropriate manipulation method but may not have had sufficient motor capability to appropriately manipulate the rake to obtain the reward, leading to a failure to obtain the reward at the beginning of the training. Analysis of the direction in which the tool is manipulated would enable the detection of trials in which the subject understood the appropriate direction in which to move the tool to obtain the reward but was not successful because of insufficient motor ability.

A previous study reported that wild chimpanzees exhibit handedness at the group level depending on the type of tool-use^25^. Wild chimpanzees are significantly more left-handed for termite-fishing, but they are significantly more right-handed for nut-cracking when using anvil and hammer stones^25^. In experimental settings, some studies have reported that human infants and common marmosets exhibit handedness at the group level depending on the direction of tool manipulation required in each experimental situation^8, 21, 26^. When older human infants grasp a single tool, they use the hand contralateral to the position of the target relative to the tool to manipulate the tool^8, 26^. In contrast, younger infants are more influenced by their hand preference than are older infants when grasping a tool^8, 26^. A previous study using common marmosets reported that the subjects usually used their hand contralateral to the position of the reward relative to the tool to manipulate it^21^. A previous study in rats investigated the dominant body parts for tool use^27^. In this study, rats were required to choose the more appropriate of two tools to obtain food beyond their reach in an experimental setting. These two tools were placed side by side around the centre of the same experimental apparatus, and rats could obtain food by pulling the appropriate tool perpendicularly to themselves. The appropriate tools were placed on the left side in half of the sessions and on the right side in the remaining sessions; rats chose the appropriate tool in most sessions in the final stage of training and during testing. This previous study reported that there were dominant body parts for each rat but that dominant parts were not observed at the group level. In other words, rats used one particular body part (left paw, right paw, or mouth) to pull tools in most of the sessions, and the body parts used were independent of the position of the tools. However, in this previous study, the rats only had to pull the tools perpendicularly to obtain food. Thus, this study never investigated whether rats exhibited preferences in which body part they used for tool manipulation in relation to different situations. No previous research in rodents, including degus, has examined whether there are dominant body parts for tool use at the group level that correlate with different situations.

In the present study, we trained rats to obtain food beyond their reach by manipulating a rake-shaped tool under different conditions. They could use both the combined position of the rake and food in the experimental apparatus and the position of the food relative to the rake as discriminative stimuli to determine how to manipulate the rake in the correct direction. After training, rats were examined to determine if they could manipulate the rake in the direction of the food under a condition in which they could use only the position of the food with respect to that of the rake as a discriminative stimulus. The tool and food were placed on the side of the experimental apparatus during training but were placed at the centre of the apparatus in the subsequent test. We examined whether rats could be trained to manipulate a tool to obtain food in a situation in which they could not obtain the food just by pulling the tool perpendicularly to themselves (Purpose 1). In addition, we examined whether rats could use only the position of the food in relation to the tool as a discriminative stimulus to manipulate the tool in the direction of the food during testing after undergoing training in which they could use both the positions of the tool and the food in the experimental apparatus and the position of the food in relation to the tool as stimuli (Purpose 2). Then, we analysed the direction of tool manipulation to examine whether rats understood the appropriate way in which to manipulate the tool to obtain the food. Moreover, we examined whether there was a group-level dominant body part for tool use in the rats depending on the situation when they were required to use a single tool (Purpose 3).

## Results

### Rake-manipulation training

**Figure 1:**
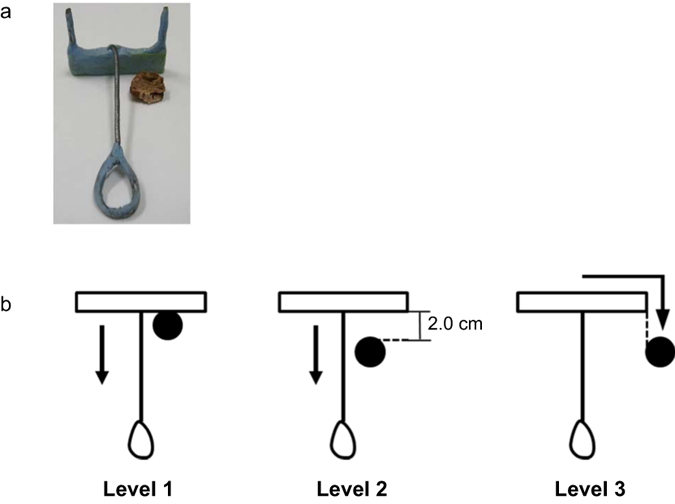
The rake-shaped tool and food reward and the arrangements of rake and reward in Levels 1, 2, and 3 of the rake-manipulation training. (**a**) The rake-shaped tool had a rectangular blade and a handle. The handle was made of wire and resin for dental use and was glued to the blade centre of the rake. The food reward was an eighth of a piece of chocolate flavoured loop cereal. (**b**) Each black circle indicates the position of the reward. Each black arrow indicates an example of the movement required to obtain the reward at each level.

**Figure 2:**
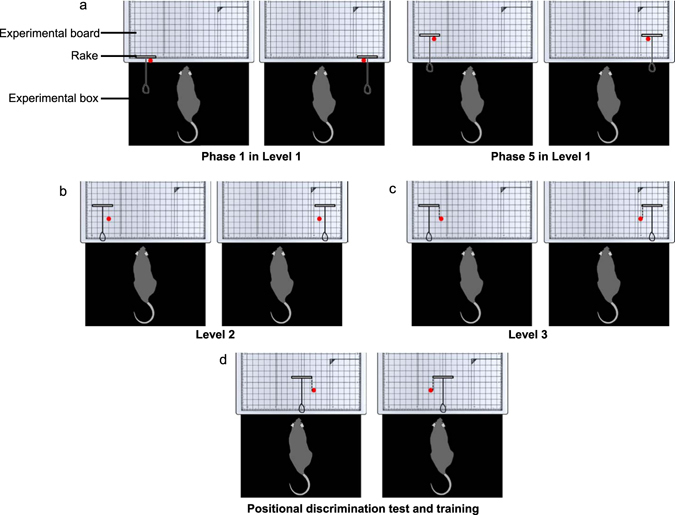
The arrangements of the rake and reward during rake-manipulation training and the positional discrimination test. Each red circle indicates the position of the reward. (**a–c**) In the rake-manipulation training (Levels 1–3), the rake was placed either on the left or right side of the experimental board. (**d**) In the positional discrimination test and training, the rake was placed at the centre of the experimental board, and the reward was placed on the left or right side of the rake. We conducted the training after the test using the same procedure.

**Figure 3:**
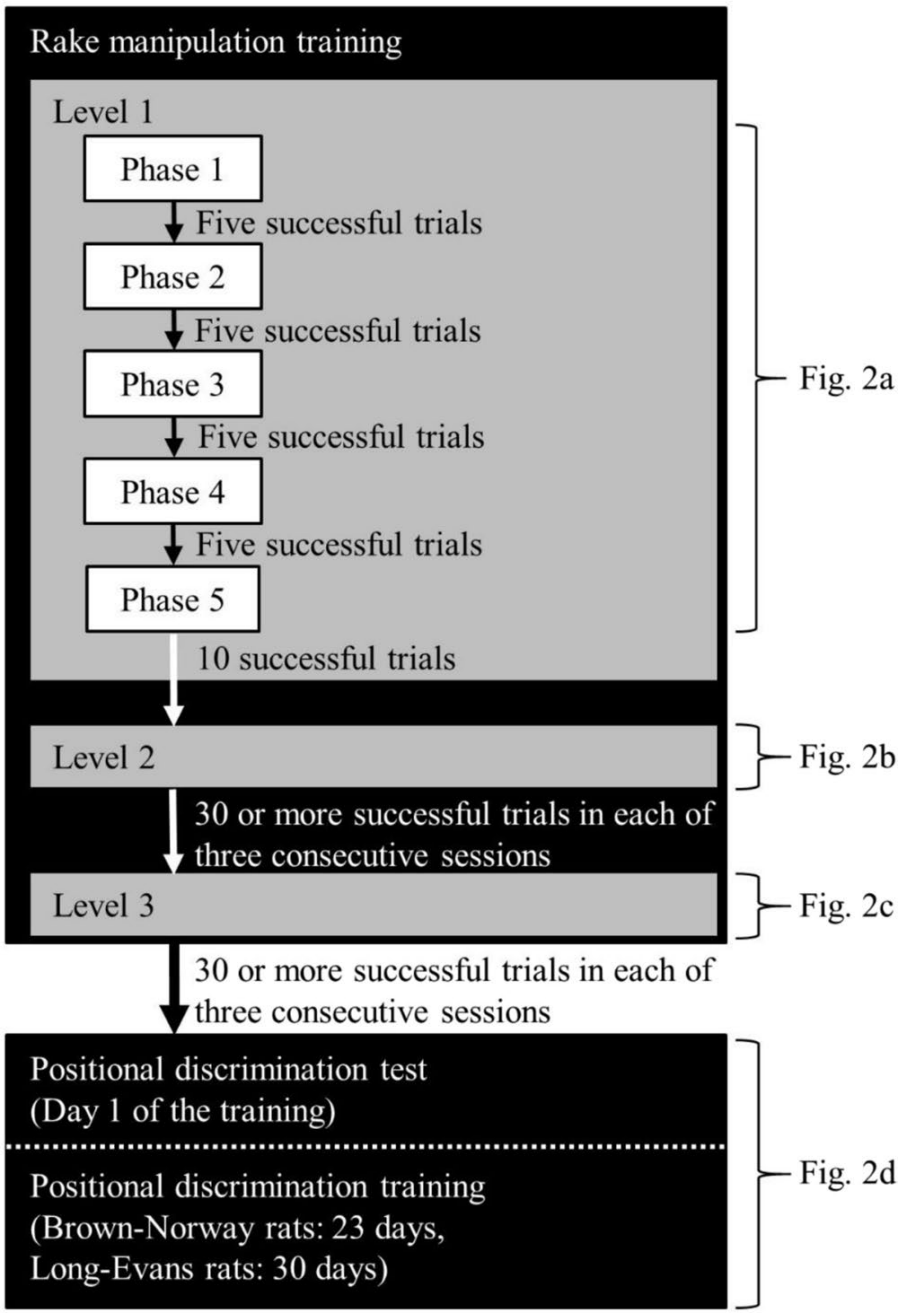
Flow chart of the rake-manipulation training and the positional discrimination test and training. The positional discrimination test corresponded to Day 1 of the positional discrimination training. Each daily experimental session consisted of 40 trials throughout this experiment.

We trained four Brown-Norway rats (subject numbers: BN1–BN4) and four Long-Evans rats (subject numbers: LE1–LE4) to use a rake-shaped tool (Fig. 1a) to obtain a food reward placed beyond their reach using a modified version of the tool-use task utilized in studies on degus^24^. In short, rake-manipulation training consisted of three levels (Levels 1–3, Fig. 1b), and we increased the distance between the rake and the reward at each level. We placed both the rake and reward on the side of the experimental apparatus during training and set the achievement criterion for each level (see Figs 2a–c, and 3 and Methods).

On the first day of rake-manipulation training (the first day of the training), a Long-Evans rat (LE3) sniffed the reward in contact with the surface of the blade of the rake, held the reward in its mouth while touching the handle of the rake and obtained the reward in the second trial (Phase 1). In the first four trials of Phase 3, even if the rat stretched out its paws for the reward and its head pushed the rake forward, the rat did not try to obtain the reward because the reward did not move towards the rat. In the following trials of this phase, when the rat stretched out its paw for the reward, it touched the handle of the rake and pulled the rake towards itself. This allowed it to learn to use the rake as a tool to obtain the reward. All rats developed the ability to use the rake to obtain the reward following this step-by-step protocol (Supplementary video S1).

**Figure 4:**
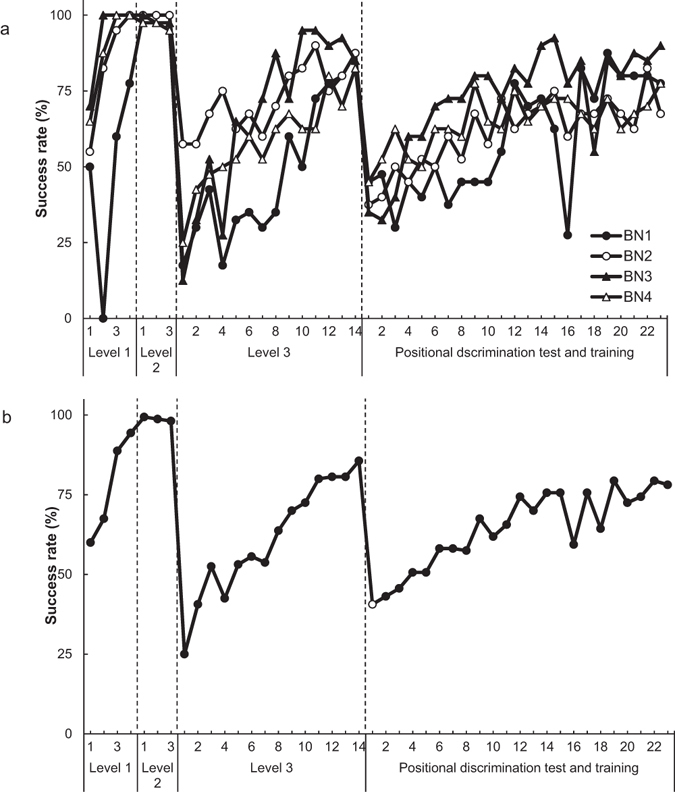
Individual and mean success rates of Brown-Norway rats (BN1–BN4) at each level of rake-manipulation training and in the positional discrimination test and training. (**a**) Individual success rates. (**b**) Mean success rate. The white circle indicates the mean success rate in the positional discrimination test.

**Figure 5:**
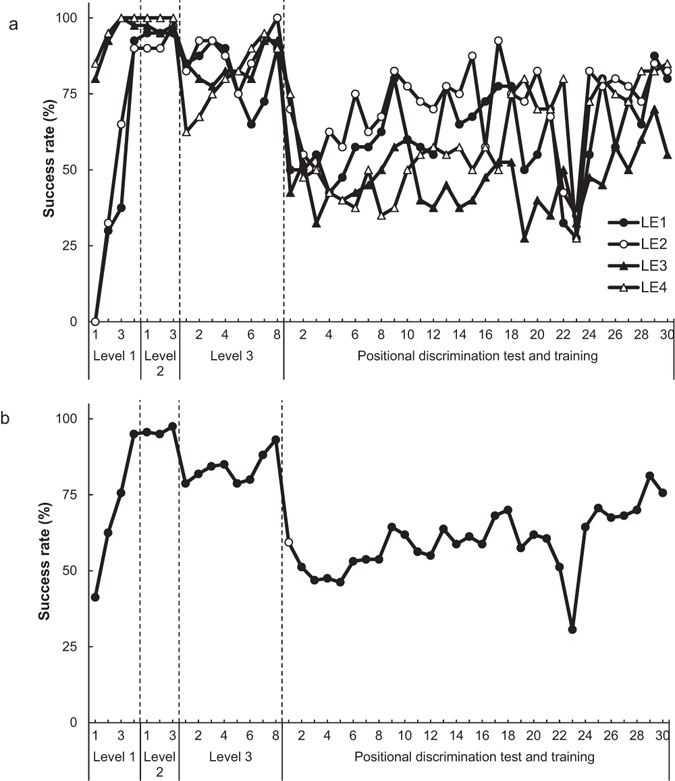
Individual and mean success rates of Long-Evans rats (LE1–LE4) at each level of rake-manipulation training and the positional discrimination test and training. (**a**) Individual success rates. (**b**) Mean success rate. The white circle indicates the mean success rate in the positional discrimination test.

We calculated the daily success rates at Levels 1, 2 and 3 by dividing the number of trials in which each rat obtained the reward within 60 s (the number of successful trials) by the number of all trials (40 trials per day) (Figs 4 and 5). At Level 1, the first success of each rat took between one and 18 trials on the first day and between 10 and 26 trials on the second day. At Level 2, all rats obtained the reward successfully in most of the trials (Supplementary video S2). The success rate of Brown-Norway rats decreased (Supplementary videos S3 and S4) significantly from last day of Level 2 to the first day of Level 3 (*t* (3) = 10.64, *p* < 0.01). In contrast, Long-Evans rats maintained a relatively high success rate from the last day of Level 2 to the first day of Level 3 (*t* (3) = 2.96, *n. s*.). The number of days required to meet the criterion for success at each level was 4 (both strains) at Level 1, 3 (both strains) at Level 2 and 8–14 (Brown-Norway rats: 14; Long-Evans rats: 8) at Level 3.

### Positional discrimination testing and training

In the positional discrimination test, we tested whether rats could manipulate the rake according to the position of the reward without additional training even if the position of the reward had been changed significantly (Purpose 2). We placed the rake at the centre of the experimental board and placed the reward on either the left or right side of the rake (Figs 2d and 3). Thus, rats could use only the position of the reward in relation to the rake as a discriminative stimulus to determine the correct direction in which to manipulate the rake (Figs 2d and 3, Supplementary video S5).

We calculated the mean success rates of Brown-Norway rats and Long-Evans rats in the positional discrimination test. To compare the success rates on the last day of Level 3 and the day of the test, we performed a paired *t*-test, with day as a within-subject factor. In Brown-Norway rats, the success rate decreased significantly from the last day of Level 3 to the day of the positional discrimination test (*t* (3) = 14.70, *p* < 0.001, Fig. 4). Likewise, in Long-Evans rats, the rate decreased significantly from the last day of Level 3 to the day of the test (*t* (3) = 4.52, *p* < 0.05, Fig. 5).

**Figure 6:**
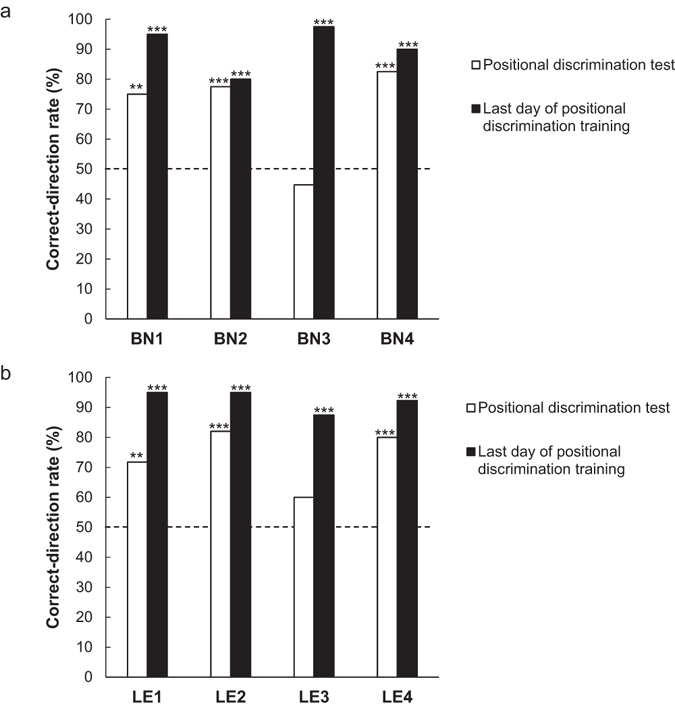
Correct-direction rates in the positional discrimination test and on the last day of the positional discrimination training for each Brown-Norway rat (BN1–BN4) and Long-Evans rat (LE1–LE4). (**a**) Correct-direction rates in each Brown-Norway rat. (**b**) Correct-direction rates in each Long-Evans rat. The broken line indicates the chance level (**p < 0.01, ***p < 0.001).

We also analysed whether rats manipulated the rake towards the reward (in the correct direction) or not (in the incorrect direction) in the positional discrimination test and compared the number of trials in which the rake was moved in either direction in this test (total 40 trials). We calculated the correct-direction rate of rake manipulation by dividing the number of trials in which each rat manipulated the rake towards the reward (the number of correct-direction trials) by the number of trials in which each rat manipulated the rake in either direction. The correct-direction trials included trials in which the rat manipulated the rake in the correct direction but failed to obtain the reward. Using binomial tests, we compared the number of correct- and incorrect-direction trials for each rat. This analysis revealed that three out of the four Brown-Norway rats manipulated the rake in the correct direction significantly more frequently than in the incorrect direction in the positional discrimination test (BN1: *p* < 0.01; BN2: *p* = 0.001; BN3: *n. s*.; BN4: *p* < 0.001, Fig. 6a). Likewise, three out of the four Long-Evans rats manipulated the rake in the correct direction significantly for frequently in this test (LE1: *p* < 0.01; LE2: *p* < 0.001; LE3: *n. s*.; LE4: *p* < 0.001, Fig. 6b). We performed the same analyses of manipulation direction for the last day of the positional discrimination training using binomial tests. This analysis revealed that all the Brown-Norway rats and Long-Evans rats manipulated the rake in the correct direction significantly more frequently on the last day of training (all the rats: *p* < 0.001, Fig. 6). To compare the correct-direction rates on the day of testing and the last day of positional discrimination training, we performed a paired *t*-test, with day as a within-subject factor. The rate increased significantly from the day of the test to the last day of training (*t* (7) = 3.59, *p* < 0.01).

**Figure 7:**
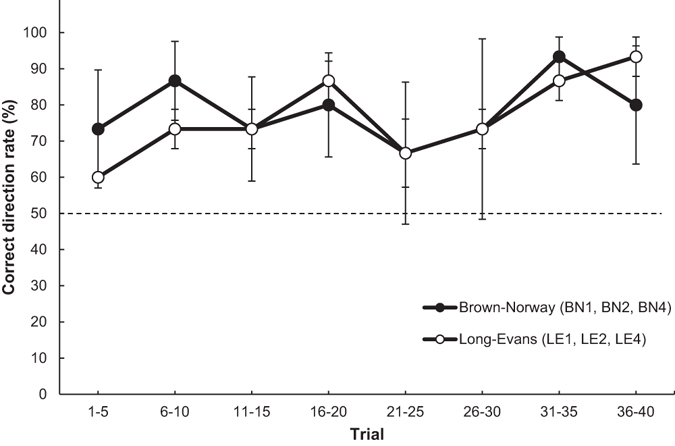
Changes in the mean correct-direction rate for rake manipulation in the positional discrimination test for six rats (subject numbers: BN1, BN2, BN4, LE1, LE2 and LE4) that could manipulate the rake in the correct direction. The broken line indicates the chance level, and error bars indicate standard errors.

We further analysed whether the rats (BN1, BN2, BN4, LE1, LE2 and LE4) that showed a significantly higher rate of manipulating the rake in the correct direction also manipulated it correctly from the beginning of the session in the test. The daily sessions (40 trials) were divided into eight blocks to calculate the mean correct-direction rate of rake manipulation, with each block consisting of five trials. We performed a two-way analysis of variance (ANOVA), with trial block as a within-subject factor and strain (i.e., Brown-Norway, Long-Evans) as a between-subject factor. The ANOVA revealed no significant main effect for trial block (Huynh-Feldt correction; *F* (7, 28) = 1.148, *n*. *s*.) or for strain (*F* (1, 4) = 0.174, *n*. *s*.) (Fig. 7).

In the following positional discrimination training, we tested whether the success rate could be increased by training (Purpose 1). We repeated the procedures described for the positional discrimination test. Two out of the four Brown-Norway rats (BN1 and BN3) reached the same criterion as in the rake-manipulation training in 23 days, and two out of the four Long-Evans rats (LE2 and LE4) reached the criterion in 30 days. To compare the success rates on the day of the positional discrimination test and on the last day of the positional discrimination training, we performed a paired *t*-test, with day as a within-subject factor. In both Brown-Norway and Long-Evans rats, the success rate on the last day of training was higher than that in the test (Brown-Norway rats: *t* (3) = 6.40, *p* < 0.01; Long-Evans rats: *t* (3) = 3.52, *p < *0.05). The mean success rates of both strains in the last three days of training exceeded 75% (Brown-Norway rats: 77.29%; Long-Evans rats: 75.63%). In short, four out of the eight rats reached the criterion in training, and the success rate in each strain increased significantly from the test to the last day of the training. We replaced the sliding door of the experimental box with a door with a rectangular hole in the lower portion on Day 22 for two Long-Evans rats (LE1 and LE2), Day 23 for all the Long-Evans rats and Day 16 for one Brown-Norway rat (BN1). This temporary replacement decreased the success rates on these days, but the rates recovered quickly following the reintroduction of the door without a hole.

### Body parts used for pulling the rake

We analysed the behaviour of rats in video records from the last day of Level 2, Day 1 and the last day of Level 3 of rake-manipulation training, and the day of the positional discrimination test (Purpose 3). In general, all rats used their left or right paws for the longest duration (see Supplementary Fig. 1). On these four days, only one rat (BN3) used its mouth in a few trials. Trials in which the rat did not touch the rake with their paws or mouth were excluded from analysis.

**Figure 8:**
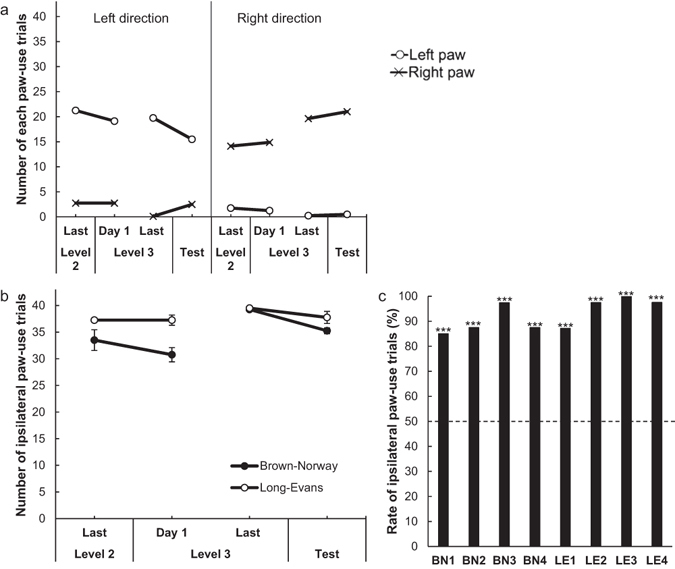
The results of analyses of whether the rats used their left or right paws in the rake-manipulation training and the positional discrimination test. (**a**) The mean number of trials in which rats manipulated the rake to the left or right and used their left or right paw for the longest duration during training and the test. (**b**) The mean number of ipsilateral paw-use trials during training and the test in Brown-Norway rats and Long-Evans rats. (**c**) The individual rates of ipsilateral paw-use trials during the test. The broken line indicates the chance level (***p < 0.001).

We conducted an additional analysis to determine whether rats used their left or right paws when they manipulated the rake in the left or right direction on the last day of Level 2, Day 1 and the last day of Level 3 in the rake-manipulation training, and the day of the positional discrimination test. We analysed the data from both strains together. In half of the sessions (20 trials) at Level 3 of the training and during the test, rats could obtain the reward by manipulating the rake to the left and in the other half (20 trials) by manipulating the rake to the right. Rats used their left paw more frequently than their right paw when they manipulated the rake to the left, and they used their right paw more frequently than their left paw when they manipulated the rake to the right (Fig. 8a, see Supplementary Fig. 2 for individual results). In short, rats tended to use the paw ipsilateral to the direction of rake manipulation more frequently than their contralateral paw. We performed a one-way ANOVA to compare the number of ipsilateral paw-use trials during training and testing, with day as a within-subject factor. The ANOVA revealed a significant main effect of day (*F* (3, 21) = 6.67, *p* < 0.01, Fig. 8b). A Bonferroni-corrected multiple comparisons test indicated that the number of ipsilateral paw-use trials increased from Day 1 to the last day of Level 3 (*p* < 0.05) and decreased from the last day of Level 3 to the test day (*p* < 0.05). In addition, we calculated the rate of ipsilateral paw-use trials in the test by dividing the number of ipsilateral paw-use trials by the number of trials in which the rats manipulated the rake in either direction. We excluded trials from this calculation where the rat flipped the rake out of reach before pulling the rake 1.0 cm towards itself (two trials for BN3 and one trial each for LE1 and LE2). In the rake-manipulation training, the sizes of the spaces for the movement of their left and right paws were not equal because the rake was placed on the left or right side of the experimental board. On the other hand, in the positional discrimination test, the sizes of the spaces for the movement of their paws were equal because the rake was placed at the centre of the board. Using binomial tests, we compared the number of ipsilateral paw-use and contralateral paw-use trials in the test for each rat. This analysis revealed that all rats used their paw ipsilateral to the direction of rake manipulation significantly more frequently than their contralateral paw in the test (Brown-Norway rats: BN1: *p* < 0.001; BN2: *p* < 0.001; BN3: *p* < 0.001; BN4: *p* < 0.001; Long-Evans rats: LE1: *p* < 0.001; LE2: *p* < 0.001; LE3: *p* < 0.001; LE4: *p* < 0.001, Fig. 8c).

### The position of the rats’ noses at the time of the first touch on the rake

We analysed the position of the rats’ noses the first time they touched the rake with their left or right paws in each trial based on the video records from the last day of rake-manipulation training and from the positional discrimination test (see SI 1 for details).

On the last day of rake-manipulation training, all of the rats had their noses on the side of the handle of the rake opposite the reward (the position closer to the periphery of the experimental board) rather than on same side as the reward (Supplementary Figs 3 and 4), and they obtained the reward successfully in most of these trials. In the positional discrimination test, the noses of the two Long-Evans rats (LE2 and LE4) that showed high success rates (LE2: 70%; LE4: 75%) and high correct-direction rates were located on the side of the handle opposite that of the reward when they obtained the food successfully (Supplementary Fig. 6). In contrast, the noses of the two rats (BN3 and LE3) that showed low success and correct-direction rates were located to the left of the handle in most of their successful trials (Supplementary Figs 5 and 6). For the other four rats (BN1, BN2, BN4 and LE1), which showed lower success rates and but still had high correct-direction rates, there was not a difference between the number of trials in which their noses were located on the same or the opposite side of the rake handle as the reward for both successful and failure trials.

## Discussion

In this study, we revealed that rats learned to use a tool to obtain food beyond their reach in an experimental situation in which they could not obtain the food just by pulling the tool perpendicularly to themselves. Rats learned to manipulate the tool in the direction of the reward before pulling it and successfully obtain the reward during positional discrimination training. Previous studies reported that macaque monkeys, common marmosets, and degus learned to manipulate a tool to obtain food in experimental settings similar to those of the present study^10–19, 21–24^. However, we revealed for the first time that rats can also accomplish this task. Thus, the current study expands the range of species that can manipulate tools according to the position of a food reward.

We conducted a positional discrimination test in which rats could use only the position of the food in relation to the tool as a discriminative stimulus, after the rake-manipulation training, in which they could use both the position of the combined tool and food within the experimental apparatus and the position of the food in relation to the tool as stimuli. The test results show that rats were able to manipulate the tool in the direction of food. We propose using direction as a new index for understanding of tool manipulation, which has thus far been overlooked in previous studies^5–8, 10–19, 21–24^. This index would be useful in examining whether rats understand the appropriate manipulation of the tool to obtain the food. The success rate decreased from the last day of Level 3 of the rake-manipulation training to the day of the positional discrimination test. However, six out of the eight rats manipulated the rake in the direction of the reward significantly more frequently than in the incorrect direction during the test. This suggests that rats understood that the reward could be moved towards themselves via contact with the blade of the rake and understood the appropriate direction in which to manipulate the rake to obtain the reward. On the last day of the positional discrimination training, the correct-direction rates of rats (BN2 and LE3) were the lowest in each strain, and these two rats showed the lowest success rates. This supports the idea that success was the result of manipulating the rake in the correct direction. It is also possible that the rats simply learned that they could obtain the reward by using the position of the reward as a discriminative stimulus and manipulating the rake toward the reward. The rats were trained to manipulate the rake in the direction of the reward at Level 3 of rake-manipulation training, and they may have simply manipulated the rake in the direction of the reward in a similar manner by using the position of the reward as a discriminative stimulus in the test. It cannot be concluded from this study which interpretation is correct, and this controversy was not fully settled in a previous study using rats^27^.

We examined whether there was a group-level dominant body part for tool use in rats that depended on the situation. There was a tendency for a dominant body part for tool use at the group level that depended on the direction of rake manipulation. Rats tended to use their paw ipsilateral to the direction of manipulation during rake-manipulation training. However, they used their ipsilateral paw less frequently in the subsequent positional discrimination test than on the last day of training. Nevertheless, for all rats, the number of trials in which they used their ipsilateral paw was above the chance level (50%) during the test (see Fig. 8c), indicating that rats prefer using their ipsilateral paw when manipulating a single tool. It could be that rats used their ipsilateral paw because they preferred abduction to adduction when making an arc to manipulate the rake. However, another possibility is that the relative position of the rake and reward in the experimental apparatus during training had an effect on the tendency of which body part was used during the positional discrimination test. During training, we placed both the rake and reward at the periphery (either left or right) of the experimental board, with the reward located closer to the centre than the rake. Thus, it is possible that rats moved their ipsilateral paws more easily than their contralateral paws during training, and they simply transferred their rake-manipulation method from training to the test. No tendency was found at the group level regarding which body part was used to pull tools in a previous study that required rats to choose between two tools^27^, but a tendency at the group level in rats was observed for the first time in the present study. However, this tendency may have been caused by the training procedure; thus, further investigation using different experimental procedures is needed to clarify these results.

What did influence the performance of the rats in this experiment? We used rats from two different strains as subjects. At Level 3 of rake-manipulation training, Brown-Norway rats took longer to meet the criterion for success than Long-Evans rats. This result could have been caused by the difference in body weight between the strains. Long-Evans rats were approximately 110 g heavier than Brown-Norway rats on the last day of free-feeding. The difference in their weight may have influenced the ease of manipulating the rake laterally, potentially leading to Long-Evans rats learning to manipulate the rake successfully in less time than Brown-Norway rats. Other influences related to the differences between each strain on their tool-use performance should be addressed in future studies.

Did the position of the rats relative to the rake and the reward at the start of each trial have some influence on their performance? On the last day of rake-manipulation training, all rats obtained the reward successfully in most of the trials, and their noses were located on the side of the rake handle opposite that of the reward (the position closer to the periphery of the experimental board) at the starting time. In the subsequent positional discrimination test, the noses of the two rats that most frequently manipulated the rake in the direction of the reward and obtained the reward were located on the side of the handle opposite that of the reward. For the other rats, this tendency was observed in each test. Thus, it could be that the rats had to move to the side of the handle of the rake opposite the reward and dynamically manipulate the rake in the direction of the reward to successfully obtain it in most trials. However, further investigation in other experimental settings is needed to generalize this influence of the relative position of subjects to the tool and the reward on their performance.

Recently, many studies have attempted to shed light on the neural mechanisms underlying tool-use behaviour in humans^28^. However, the neural mechanism underpinning human tool-use behaviour remains unclear^29^, and additional research is needed. Recent studies have investigated the neural mechanism of tool-use behaviour in macaque monkeys^10, 13, 15–17, 20^, common marmosets^22^ and degus^23^. A study on patients with left brain damage suggests that the parietal lobe contributes to tool-use behaviour^30^. A study on Japanese monkeys (*Macaca fuscata*) suggests that the intraparietal region, basal ganglia, presupplementary motor area, premotor cortex and cerebellum contribute to tool-use behaviour^16^. Using positron emission tomography, this study showed that tool-use-related activities involved a significant increase in cerebral blood flow in these areas^16^. However, studies using rats would be able to employ various experimental manipulations, including microinjections of drugs into specific areas of the brain and electrocautery lesions made in specific areas, that are not practical for use with humans, nonhuman primates, and other rodents^30^. Thus, we propose the rat as an animal model to investigate the behavioural and neural mechanism of tool use in animals and potentially humans.

## Methods

### Subjects

Four experimentally naïve male Brown-Norway and four male Long-Evans rats (Shimizu, Kyoto, Japan) were individually housed in wire cages. All rats were 2-months old. Brown-Norway rats weighed a mean of 185.00 g (*SD* = 2.74), and Long-Evans rats weighed a mean of 296.25 g (*SD* = 4.82) on the last day of free-feeding. During training and testing, rats were maintained at approximately 85–90% of their free-feeding weight. The animal room was maintained under a 12:12-h alternating light-dark cycle (light phase 08:00–20:00). We conducted all procedures during the light phase. All procedures and treatments were approved by the Doshisha University Animal Experiment Committee and were conducted in accordance with guidelines established by the Doshisha University Ethics Review Committee.

### Apparatus

The experiments took place in an experimental box (outer dimensions: 21.0 cm wide × 21.0 cm long × 25.6 cm high) made of transparent acrylic boards, and placed on a desk. A transparent sliding door, which the experimenter could open or close, was mounted at the front of the box. An experimental board (23.9 cm wide × 33.5 cm long), on which the tool and reward were presented, was set in front of the sliding door. Details of the experimental box have been described elsewhere^27^.

The tool was rake-shaped (3.5 cm maximum wide × 6.5 cm long × 2.9 cm maximum high; weight: 4.7 g, Fig. 1a), with a rectangular blade (3.5 cm wide × 1.1 cm high × 0.3 cm thick) and a wire handle (1.2 cm maximum wide × 6.2 cm long). The blade was made of a plastic plate covered with resin originally developed for dental use (Ostron II Blue, GC Corporation, Tokyo, Japan) to reinforce the plastic plate and prevent the rats from gnawing on it.

During training and testing, we recorded each subject’s behaviour using a video camera (Panasonic, Japan, HDC-TM30). The experimenter sat at the front of the box, observed the subject’s behaviour, and conducted the following behavioural procedures.

### Procedures

#### Habituation

Before rake-manipulation training, each rat was habituated to the food reward by receiving the chocolate flavoured loop cereals used as rewards (Ciscorn Sakusaku Ring, Nissin Cisco Co., Ltd., Osaka, Japan) in their cage.

#### Rake-manipulation training

In rake-manipulation training, the procedures used were a modified version of those used in the tool-use task for degus^24^. Training consisted of three levels, with rats trained to obtain a piece of food beyond their reach. Each daily experimental session consisted of 40 trials. An eighth of a piece of cereal was used as a reward in each trial.

At the first training level (Level 1), rats were trained to obtain the reward just by pulling the tool perpendicularly to the sliding door of the experimental box. The experimenter placed the reward between the blade of the rake and the rat, with the reward in contact with the surface of the blade (Figs 1b and 2a). The sliding door of the experimental box was kept open throughout this level so that the space between the bottom of the door and the surface of the experimental board was 1.7 cm. At the beginning of the session, the experimenter placed the rat in the box. Trials started when the experimenter placed the rake and the reward at the defined position on the board (Fig. 2a). Trials ended either when the rat had obtained the reward or after 60 s had passed. If the reward entered the box, we recorded it as a successful trial, and if the reward could not be obtained within 60 s, we recorded it as a failure trial. In Level 1, which was further divided into five phases, the distance between both the rake and reward and the rat was made increasingly longer in five phases (Figs 2a and 3). In Phase 1, the experimenter placed the rake and reward so that the distance between reward and sliding door was 0 cm. The distance was increased by 1.0 cm every time the rat reached the criterion of the previous phase. Both the rake and reward were placed on the side of the board in all phases of Level 1, alternating between the left or right side. We adopted this arrangement to promote the movement of the rake to the centre of the experimental board more easily, prompting the rats to move the rake laterally and decreasing the number of days required for training. Each arrangement was adopted in one-half of the trials on each day in pseudo-randomized order. From Phases 1 to 4, five successful trials resulted in the advancement to the next phase. In Phase 5, the rats were required to complete 10 successful trials to meet the success criterion. In Phases 1 to 5, five or 10 non-consecutive successful trials were allowed to meet each criterion. This training at Level 1 continued for each rat until it met the criterion for Phase 5. On the first day of Level 1 training, we carried out the training for Phase 5 for two Long-Evans rats (subjects LE1 and LE2) by omitting Phases 1 to 4, as they never pulled the rake on this day. Thus, we started the training at Phase 1 for these two Long-Evans rats on the next day.

At Level 2, rats were trained to pull the rake perpendicularly to the sliding door as in Level 1. The same procedure was adopted as in Level 1 except for the position of the rake, the operating condition of the sliding door, and the achievement criterion. The distance between the blade of the rake and the sliding door was 2.0 cm longer than at Level 1 (Figs 1b, 2b and 3). The rat could obtain the reward just by pulling the rake perpendicularly, as in Level 1. At the beginning of the session, the rat was placed in the box with the sliding door closed. As soon as the experimenter placed the rake and the reward at the defined position on the experimental board (Fig. 2b), the sliding door was opened (trial start). The space between the bottom of the door and the surface of the board was 1.7 cm so that the rat could touch the rake with its forelimbs or mouth. The experimenter closed the door, and the trial ended either when the rat obtained the reward or when 60 s had passed. Meeting the success criterion required each rat to achieve 30 or more successful trials in each of three consecutive sessions, and training was continued until all rats in each strain met this criterion. The same criterion was adopted at Level 3 and for the positional discrimination training.

At Level 3, rats were trained to obtain the reward by manipulating the rake in a situation in which they could not obtain the reward just by pulling the rake perpendicularly to themselves. At this level, the same procedure as in Level 2 was used, except for position of the reward. The reward was placed at a position in which, even if the rat pulled the rake perpendicularly, the blade of the rake would not touch the reward (Figs 1b and 2c). When the rake was placed on the rat’s right, the reward was placed on the left side of the rake, and vice versa. However, one Brown-Norway rat (BN4) did not meet the criterion for this level for 14 days. Its performance was comparable to that of the other three Brown-Norway rats (BN1–BN3). Thus, we carried out the following positional discrimination test for this rat (BN4) on the same day that the other three rats were tested.

#### Positional discrimination test

In this test, we examined whether rats could manipulate the rake according to reward position without added training, even if the position of the reward had been changed significantly. The experimenter placed the rake at the centre of the board, and the reward was placed on either the left or right side of the rake (Fig. 2d). In this test, the spatial arrangement of the rake and reward, in terms of the horizontal and vertical distances of the reward from the rake, were the same as in Level 3. Therefore, the rat could use only the position of the reward in relation to the rake as a discriminative stimulus and had to manipulate the rake in the direction of the reward. The daily experimental session in this test consisted of 40 trials.

#### Positional discrimination training

In this training, we continued to use the same procedures as those in the positional discrimination test (Fig. 3). This training was conducted for 23 days in Brown-Norway rats and for 30 days in Long-Evans rats. In Long-Evans rats, however, we increased the distance between both the rake and the reward and the sliding door by 1.0 cm because Long-Evans rats could almost reach the reward without the rake during the positional discrimination test. On Day 22 for two Long-Evans rats (LE1 and LE2), Day 23 for all Long-Evans rats and Day 16 for one Brown-Norway rat (BN1), we used a sliding door with a rectangular hole in the lower portion of the door. The hole allowed the rats to look at the rake and the reward directly (see SI 2 for details).

#### Data scoring and analysis

In the positional discrimination test and on the last day of positional discrimination training, when the rat manipulated the rake towards the reward, we recorded it as a correct-direction trial. Meanwhile, when the rat manipulated the rake away from the reward, we recorded it as an incorrect-direction trial. These determinations were based on whether the intersection point of the blade and the handle of the rake was on the left or right side of the centre-line of the experimental board when the rat pulled the rake 2.0 cm (i.e., to the horizontal line contacting the reward). Four trials in total were excluded from the analysis as the rats flipped the rake out of reach before pulling the rake 1.0 cm (test: two trials for BN3 and one trial each for LE1 and LE2; last day of training: one trial for LE4). We performed the same behavioural analyses regarding the direction of manipulation for the last day of Level 2, and Day 1 and the last day of Level 3 in the rake-manipulation training as were performed for the positional discrimination test.

We recorded which body part (left paw, right paw, or mouth) the rats used when they pulled the rake in each trial. We recorded whether each body part was used or not from the time when the trial started to the time when the rat obtained the reward (successful trial) or until 60 s had passed (failure trial). Here, we focused on the body part used for the longest duration in each trial. If the rat used multiple body parts in a trial, we compared the cumulative times of use for each body part and recorded the time for the body part that was used for the longest duration. For example, if a rat used its left paw for 0.50 s and right paw for 0.57 s in a trial, it was recorded as a trial in which the right paw was used for the longest duration.

Moreover, we analysed the position of the rats’ noses when they first touched the rake with their left or right paws in each trial based on the video records for the last day of rake-manipulation training and for the positional discrimination test. We conducted this analysis to investigate whether the position of the rat in relation to the rake and the reward had an influence on their success rate (see SI 1 for details).

## Electronic supplementary material

Supplementary Information

Video S1

Video S2

Video S3

Video S4

Video S5

## References

[1] St. Amant R, Horton TE (2008). Revisiting the definition of animal tool use. Anim. Behav..

[2] Bentley-Condit VK, Smith EO (2010). Animal tool use: current definitions and an updated comprehensive catalog. Behaviour.

[3] Tebbich S, Taborsky M, Fessl B, Dvorak M (2002). The ecology of tool-use in the woodpecker finch (*Cactospiza pallida*). Ecol. Lett..

[4] Boesch C, Boesch H (1990). Tool use and tool making in wild chimpanzees. Folia Primatol.

[5] Fagard J, Rat-Fischer L, O’Regan JK (2014). The emergence of use of a rake-like tool: a longitudinal study in human infants. Front. Psychol..

[6] Petkovic M, Rat-Fischer L, Fagard J (2016). The emergence of tool use in preterm infants. Front. Psychol..

[7] Rat-Fischer L, O’Regan JK, Fagard J (2012). The emergence of tool use during the second year of life. J. Exp. Child Psycol..

[8] Rat-Fischer L, O’Regan JK, Fagard J (2013). Handedness in infants’ tool use. Dev. Psychobiol..

[9] Fagard J, Rat-Fischer L, Esseily R, Somogyi E, O’Regan JK (2016). What does it take for an infant to learn how to use a tool by observation?. Front. Psychol..

[10] Hihara S (2006). Extension of corticocortical afferents into the anterior bank of the intraparietal sulcus by tool-use training in adult monkeys. Neuropsychologia.

[11] Hihara S, Obayashi S, Tanaka M, Iriki A (2003). Rapid learning of sequential tool use by macaque monkeys. Physiol. Behav..

[12] Hihara S, Yamada H, Iriki A, Okanoya K (2003). Spontaneous vocal differentiation of coo-calls for tools and food in Japanese monkeys. Neurosci. Res..

[13] Iriki A, Tanaka M, Iwamura Y (1996). Coding of modified body schema during tool use by macaque postcentral neurons. Neuroreport.

[14] Ishibashi H, Hihara S, Iriki A (2000). Acquisition and development of monkey tool-use: behavioral and kinematic analyses. Can. J. Physiol. Pharmacol..

[15] Ishibashi H (2002). Tool-use learning selectively induces expression of brain-derived neurotrophic factor, its receptor *trk*B, and neurotrophin 3 in the intraparietal multisensorycortex of monkeys. Cogn. Brain Res..

[16] Obayashi S (2001). Functional brain mapping of monkey tool use. Neuroimage.

[17] Quallo MM (2009). Gray and white matter changes associated with tool-use learning in macaque monkeys. Proc. Natl. Acad. Sci. USA.

[18] Yamazaki Y, Kurihara Y, Iriki A, Watanabe S (2009). Changes in the repertoire of tool-using behaviour in Japanese monkeys. CARLS series of advanced study of logic and sensibility.

[19] Yamazaki Y, Namba H, Iriki A (2009). Acquisition of an externalized eye by Japanese monkeys. Exp. Brain Res..

[20] Quallo MM, Kraskov A, Lemon RN (2012). The activity of primary motor cortex corticospinal neurons during tool use by macaque monkeys. J. Neurosci..

[21] Yamazaki Y (2011). Tool-use learning by common marmosets (*Callithrix jacchus*). Exp. Brain Res..

[22] Yamazaki Y (2016). Neural changes in the primate brain correlated with the evolution of complex motor skills. Sci. Rep..

[23] Kumazawa-Manita N, Hama H, Miyawaki A, Iriki A (2013). Tool use specific neurogenesis and synaptogenesis in rodent (*Octodon degus*) hippocampus. PLoS One.

[24] Okanoya K, Tokimoto N, Kumazawa N, Hihara S, Iriki A (2008). Tool-use training in a species of rodent: the emergence of an optimal motor strategy and functional understanding. PLoS One.

[25] Lonsdorf EV, Hopkins WD (2005). Wild chimpanzees show population-level handedness for tool use. Proc. Natl. Acad. Sci..

[26] Cox RFA, Smitsman AW (2006). Action planning in young children’s tool use. Dev. Sci..

[27] Nagano A, Aoyama K (2017). Tool-use by rats (*Rattus norvegicus*): tool-choice based on tool features. Anim. Cogn..

[28] Goldenberg G, Spatt J (2009). The neural basis of tool use. Brain.

[29] Bi Y (2015). The white matter structural network underlying human tool use and tool understanding. J. Neurosci..

[30] Cenci MA, Whishaw IQ, Schallert T (2002). Animal models of neurological deficits: how relevant is the rats?. Nat Rev Neurosci..

