# Supplementary material for: Tool manipulation by rats (Rattus norvegicus) according to the position of food
Source: Sci Rep. 2017 Jul 20;7:5960. doi: 10.1038/s41598-017-06308-7 (PMC5519611; doi:10.1038/s41598-017-06308-7)
Supplement: Supplementary file 1 — Supplementary Information [file 41598_2017_6308_MOESM1_ESM.pdf]

# Supplementary Materials for

## Tool manipulation by rats (*Rattus norvegicus*) according to the position of food

Authors: A. Nagano* and K. Aoyama

*Correspondence to: Akane Nagano (ekp1003@mail2.doshisha.ac.jp)

**This PDF file includes:**

Supplementary Information. SI1 to SI2

Figs. S1 to S6

**Supplementary Information**

<u>**SI 1** Analysis of the positon of the rats' noses when first touching the rake</u>

We analysed the position of the rats' noses when they first touched the rake with their left or right paws in each trial based on the video records from the last day of the rake-manipulation training and from the positional discrimination tests. For this analysis, we divided the first column into 21 areas (Area 1–Area 21) based on the squares of the experimental board as shown in the following figure. We recorded the position of the rat's nose after the trial started. Area 11 was the centre of the board.

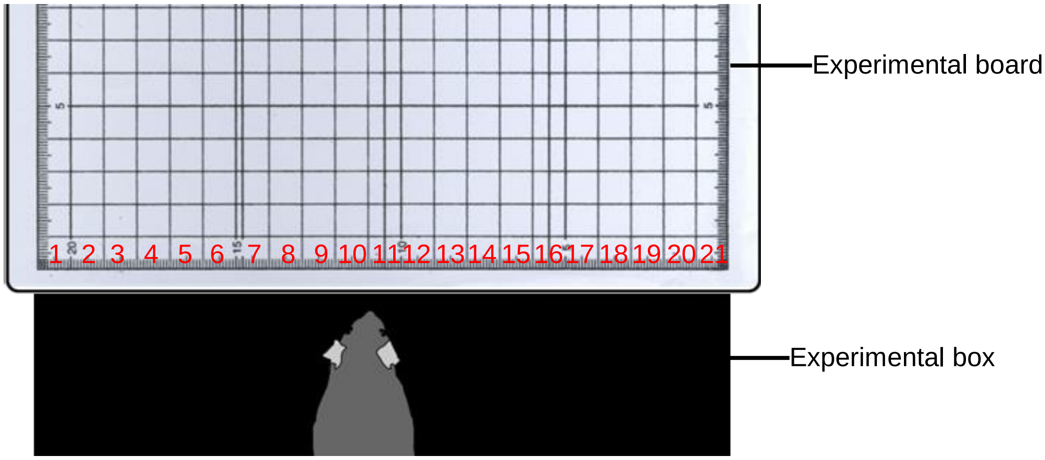


<u>**SI 2** Temporary change of the sliding door of the experimental box in the positional discrimination training</u>

On Day 22 for two Long-Evans rats (LE1 and LE2), Day 23 for all the Long-Evans rats and Day 16 for one Brown-Norway rat (BN1) of the positional discrimination training, we used a sliding door with a rectangular hole in the lower portion of the door. The hole was centred horizontally in the door. Training Day 16 for BN1 was the same day as Day 23 for LE1–LE4. On Day 22 for LE1 and LE2, the rectangular hole (4.0 cm wide × 3.0 cm high) was located 5.0 cm high on the door. On Day 23 for LE1–LE4 and Day 16 for BN1, a smaller rectangular hole (3.0 cm wide × 2.0 cm high) was located 5.0 cm high on the door.

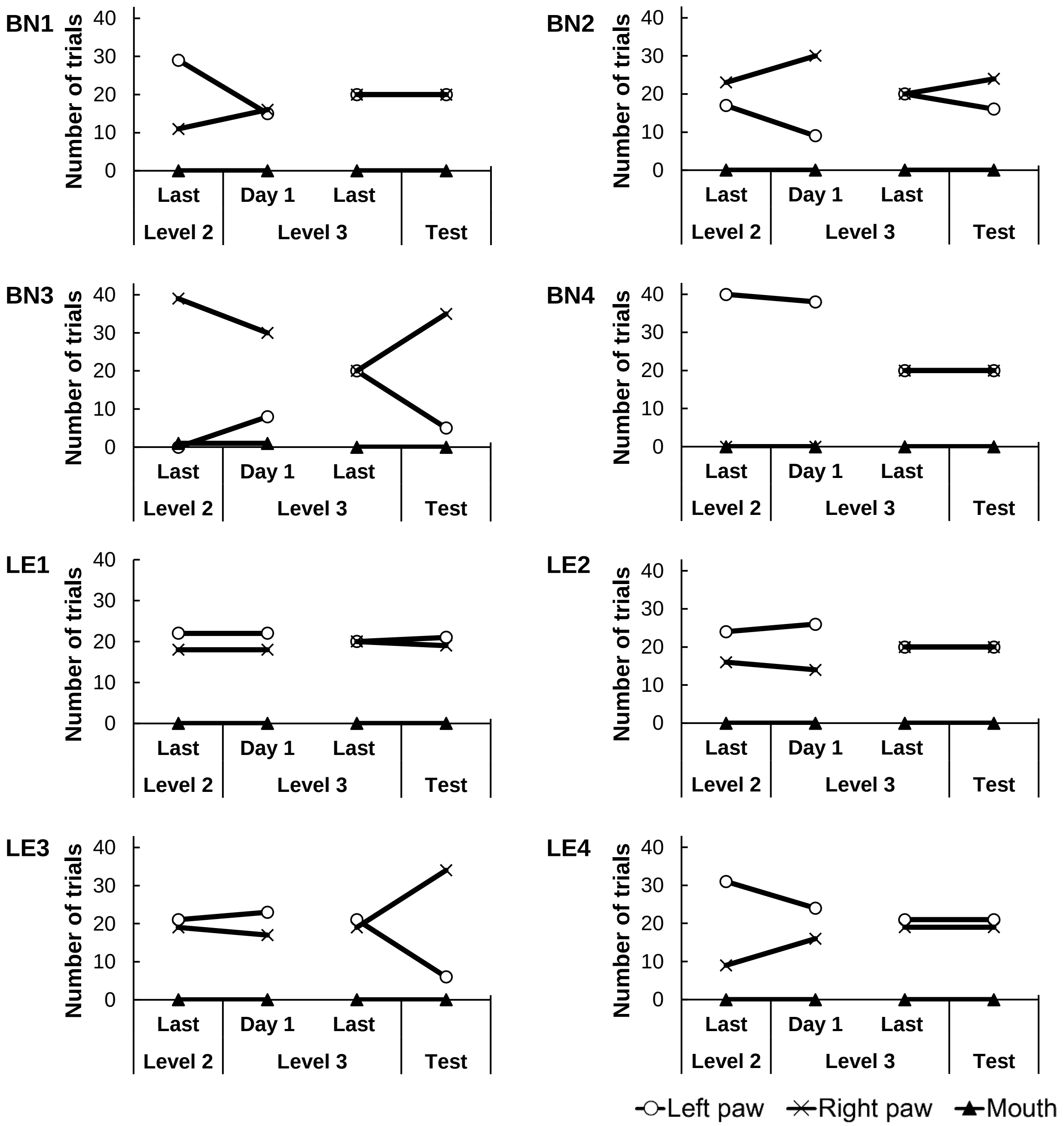


**Supplementary Figure 1.** Individual (Brown-Norway rats: BN1–BN4; Long-Evans rats: LE1–LE4) results for the number of trials and for which body part was used for the longest duration, throughout rake-manipulation training and the positional discrimination tests. Each white circle indicates the number of left paw-use trials; each cross mark, the number of right paw-use trials. Each black triangle indicates the number of mouth-use trials.

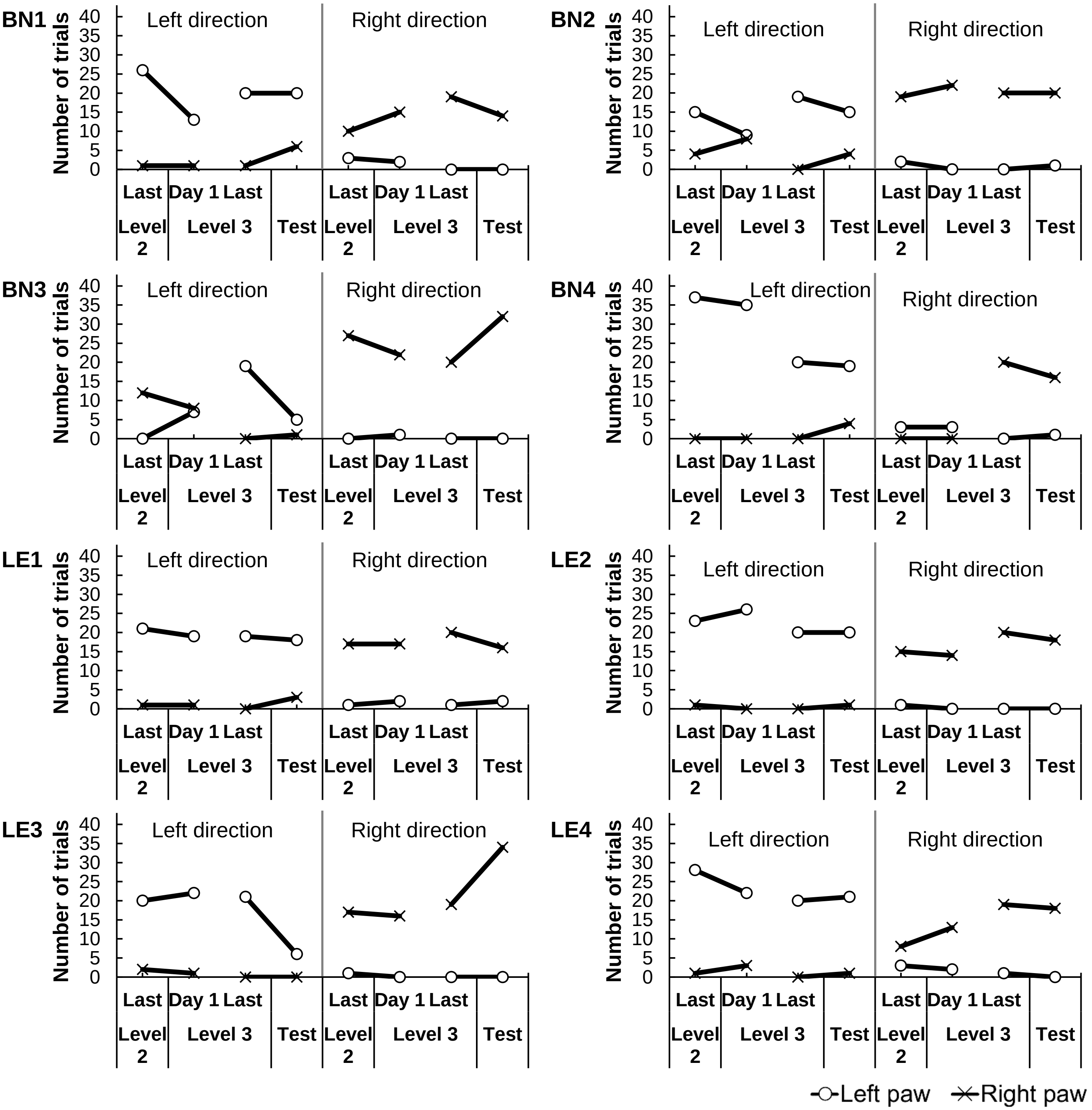


**Supplementary Figure 2.** Individual (Brown-Norway rats: BN1–BN4; Long-Evans rats: LE1–LE4) results for the number of trials in which rats manipulated the rake to the left or right with their left or right paw and with paw was used for the longest duration throughout rake-manipulation training and the positional discrimination test. Each white circle indicates the number of left paw-use trials; each cross mark, the number of right paw-use trials.

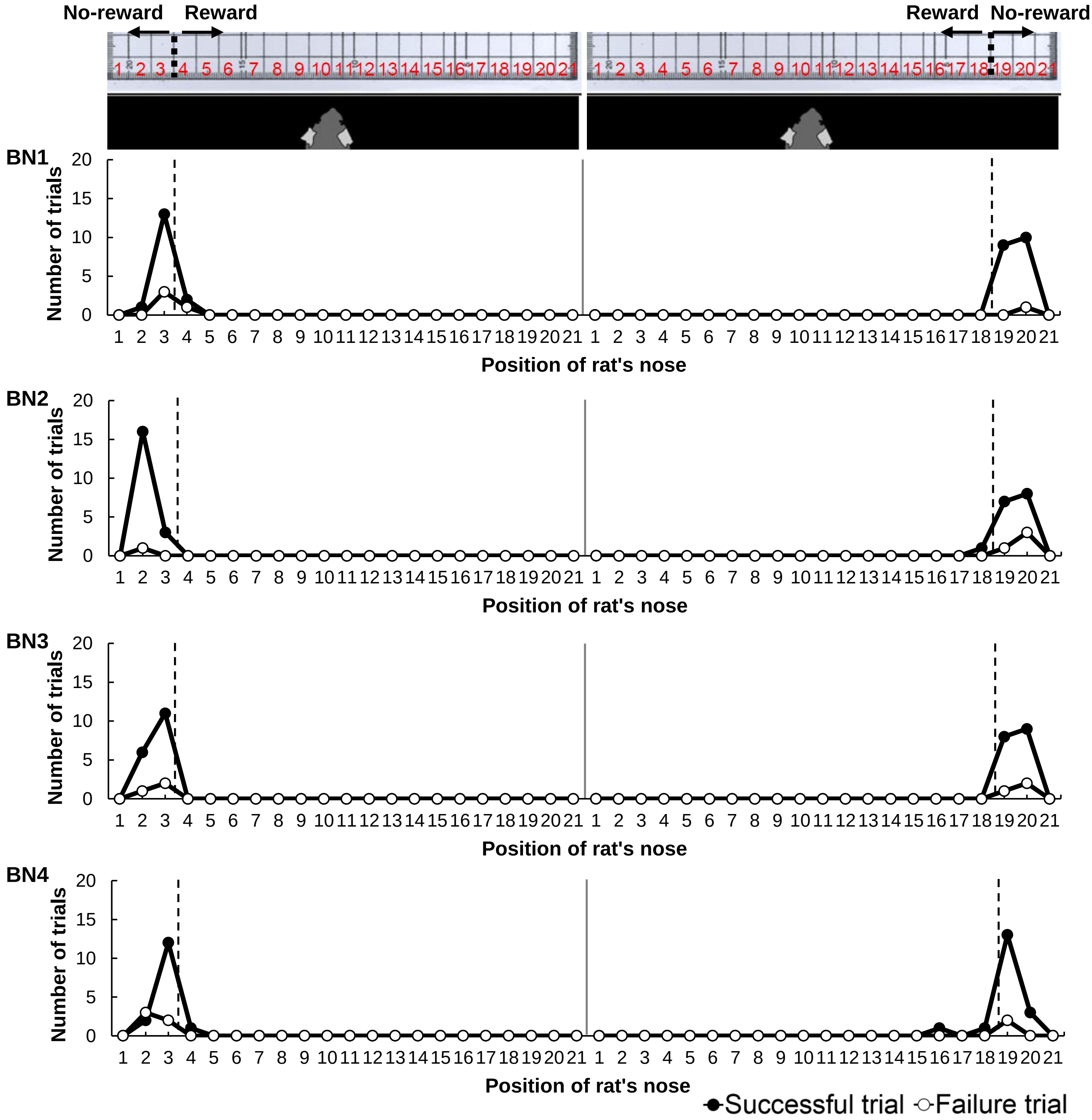


**Supplementary Figure 3.** Individual results for Brown-Norway rats (BN1–BN4) for the number of trials in which the rat's nose was located at each area in each trial on the last day of rake-manipulation training. The left panel indicates the results in which the reward was placed on the right side of the rake; the right panel, the results in which the reward was placed on the left side of the rake. Each black circle indicates the number of successful trials; each white circle, the number of failure trials. Each broken line indicates the position of the handle of the rake.

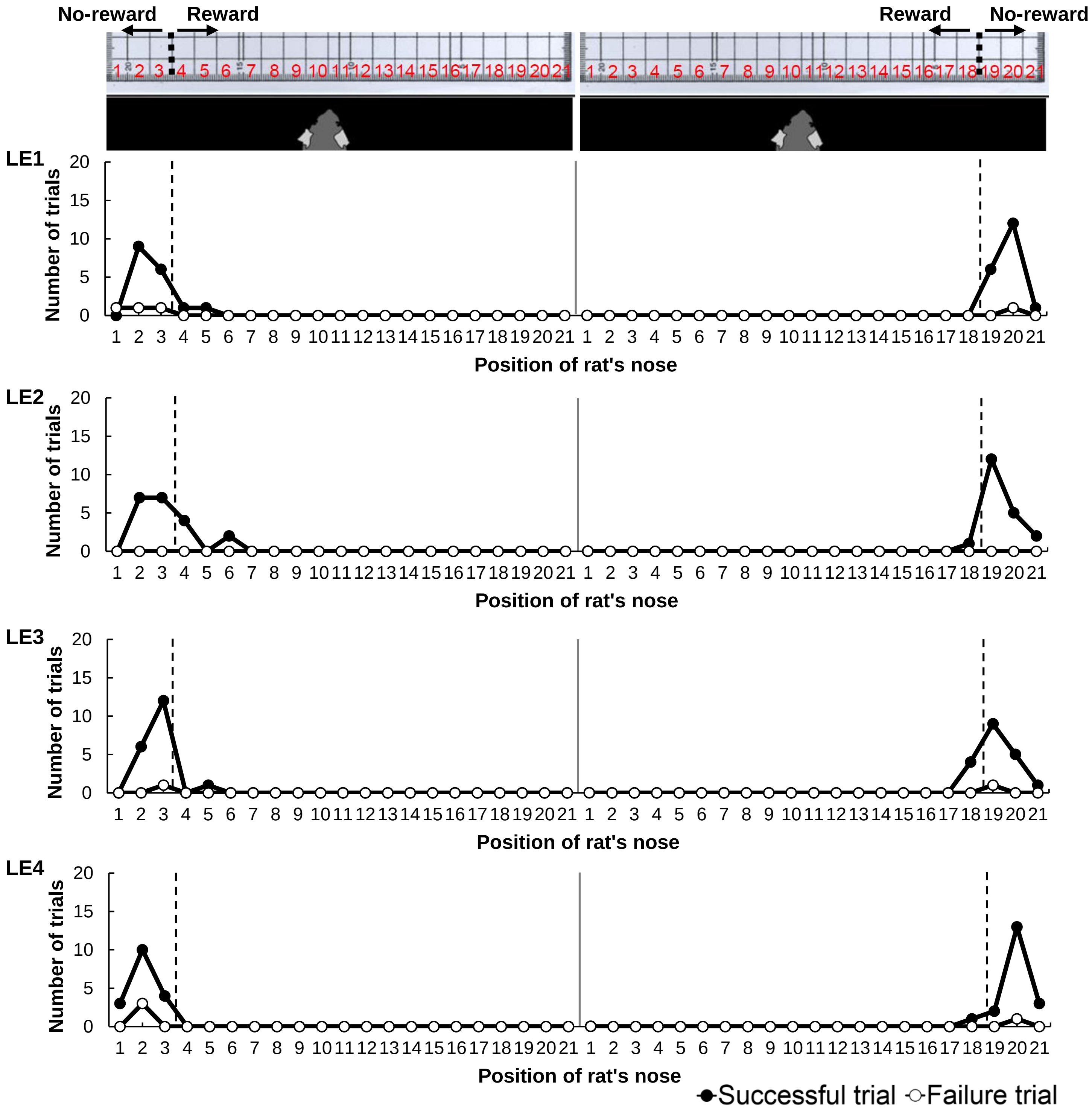


**Supplementary Figure 4.** Individual results for Long-Evans rats (LE1–LE4) for the number of trials in which the rat's nose was located at each area in each trial on the last day of rake-manipulation training. The left panel indicates the results in which the reward was placed on the right side of the rake; the right panel, the results in which the reward was placed on the left side of the rake. Each black circle indicates the number of successful trials; each white circle, the number of failure trials. Each broken line indicates the position of the handle of the rake.

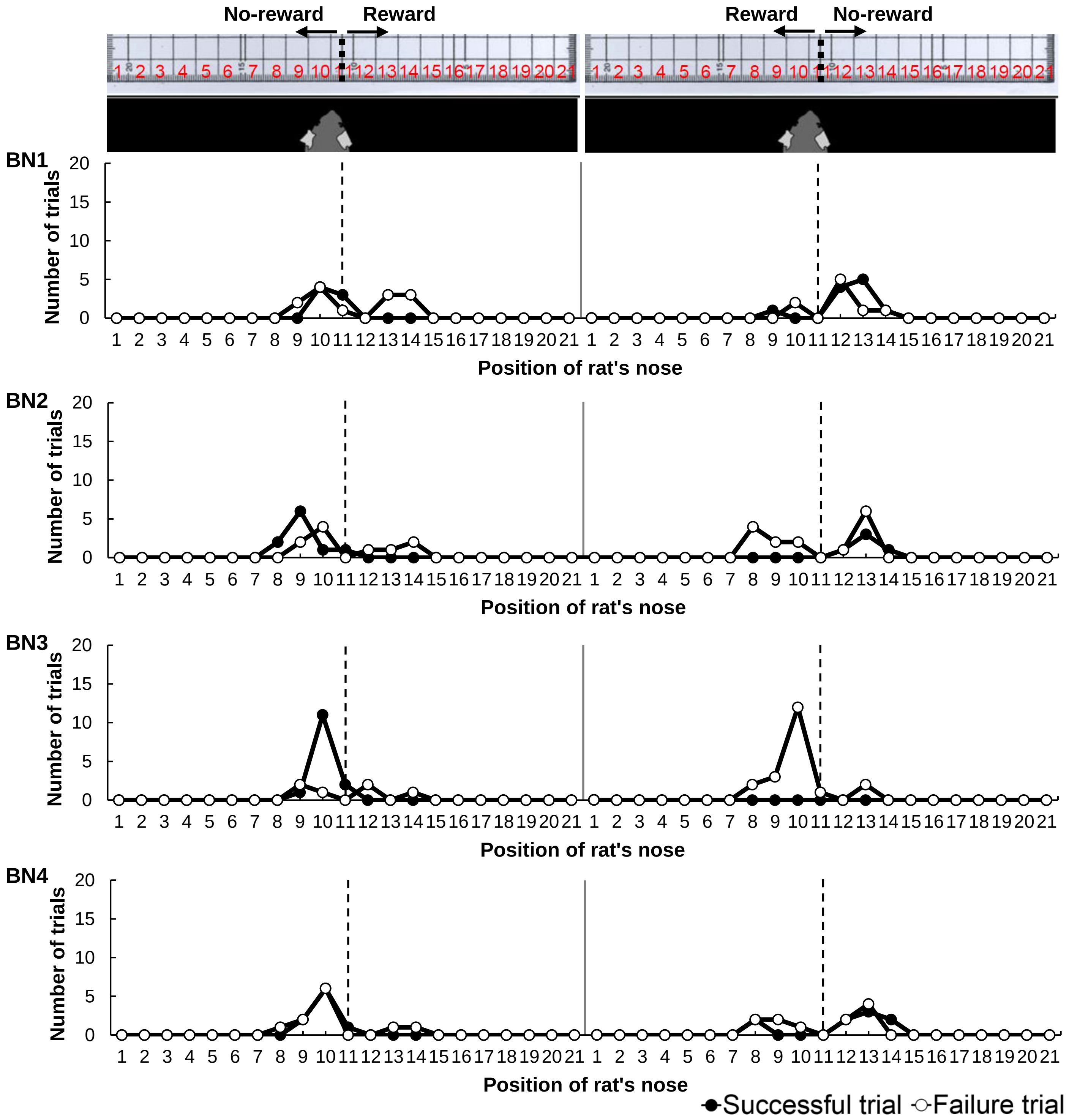


**Supplementary Figure 5.** Individual results for Brown-Norway rats (BN1–BN4) for the number of trials in which the rat's nose was located at each area in each trial in the positional discrimination test. The left panel indicates the results in which the reward was placed on the right side of the rake; the right panel, the results in which the reward was placed on the left side of the rake. Each black circle indicates the number of successful trials; each white circle, the number of failure trials. Each broken line indicates the position of the handle of the rake.

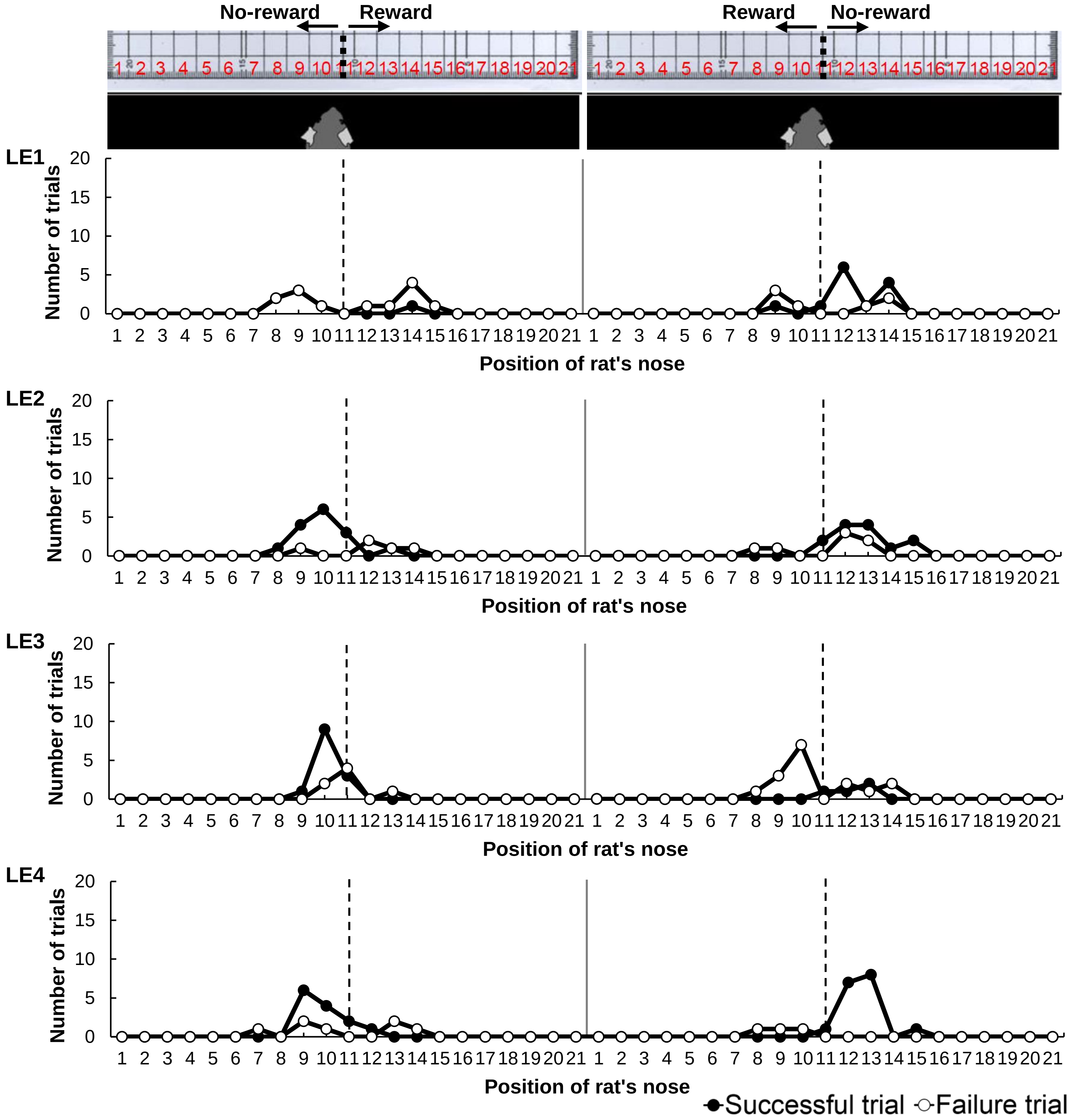


**Supplementary Figure 6.** Individual results for Long-Evans rats (LE1–LE4) for the number of trials in which the rat's nose was located at each area in each trial in the positional discrimination test. The left panel indicates the results in which the reward was placed on the right side of the rake; the right panel, the results in which the reward was placed on the left side of the rake. Each black circle indicates the number of successful trials; each white circle, the number of failure trials. Each broken line indicates the position of the handle of the rake.
